# CT-to-surgery interval and occult nodal metastasis in cN0 oral squamous cell carcinoma

**DOI:** 10.1007/s00432-026-06642-2

**Published:** 2026-10-16

**Authors:** Jannik Ketschau, Jonathan Mohr, Alex Grabenhorst, Hannes Singer, Helena Richter, Nils Krautkremer, Minli Niu, Katharina Pippich, Herbert Stimmer, Markus Graf, Klaus-Dietrich Wolff, Lucas M. Ritschl

**Affiliations:** 1 Department of Oral and Maxillofacial Surgery, School of Medicine and Health, TUM University Hospital Klinikum Rechts der Isar, Technical University of Munich, Ismaninger Strasse 22, D-81675 Munich, Germany; 2 School of Medicine and Health, Institute of Diagnostic and Interventional Radiology, TUM University Hospital Klinikum Rechts der Isar, Technical University of Munich, Munich, Germany

**Keywords:** Oral squamous cell carcinoma, Occult lymph node metastasis, Neck dissection, CT-to-surgery interval, cN0 neck, Extranodal extension

## Abstract

**Background:**

CT-based staging of the clinically node-negative (cN0) neck in oral squamous cell carcinoma (OSCC) has limited sensitivity, and occult nodal metastasis remains common. Whether the CT-to-surgery interval influences pathological nodal outcome is unclear.

**Methods:**

This retrospective study included 329 patients with primary cN0 OSCC undergoing CT staging and neck dissection (2011–2024). Associations with lymph node positivity, lymph node count, and extranodal extension (ENE) were assessed using logistic and negative binomial regression, with sensitivity analyses (quartiles, exclusion of extreme intervals, lymph node yield adjustment).

**Results:**

Median CT-to-surgery interval was 9.0 days (IQR 5.0–15.0), and 53 patients (16.1%) had lymph node metastasis. The interval was associated with lymph node positivity in univariable analysis (OR 1.031, *p* = 0.035), but not after adjustment for age and smoking (OR 1.027, *p* = 0.065); lymph node count showed a similar non-significant association (IRR 1.027, *p* = 0.068). The association with nodal positivity was not robust across interval quartiles (*p* = 0.969), after excluding intervals >30 days (OR 0.995, *p* = 0.824), or after adjustment for lymph node yield (OR 1.015, *p* = 0.334). However, patients with intervals >30 days (n = 13) had higher nodal positivity than those with shorter intervals (53.8% vs. 14.6%). The interval was not associated with ENE (*p* = 0.740), and tumor localization was not associated with nodal positivity (*p* = 0.175).

**Conclusion:**

An initially significant, unadjusted association was not robust to sensitivity testing and was confined to a small subgroup with substantially prolonged intervals. This offers some reassurance that the CT-to-surgery interval is not strongly associated with occult nodal metastasis in cN0 OSCC, while substantially delayed surgery warrants further investigation.

## Introduction

Cervical lymph node status is a powerful prognostic determinant in oral squamous cell carcinoma (OSCC). Approximately 40% of patients harbor lymph node metastases, and nodal involvement reduces survival by roughly 50%, with nodal number, size, level, laterality, and extranodal extension each contributing independent prognostic weight (Struckmeier et al. 2024; Ekanayaka and Tilakaratne 2025; Johansson et al. 2026). Beyond nodal positivity itself, the lymph node ratio has been shown to independently predict ipsilateral nodal recurrence and shorter recurrence-free survival in OSCC of the tongue and floor-of-mouth, underscoring the prognostic relevance of the extent of nodal involvement rather than positivity alone (Ketschau et al. 2026).

However, standard cross-sectional imaging is known to miss a clinically important fraction of nodal disease, which is why neck dissection rather than imaging-based observation is generally advocated for the clinically negative neck (Horvath et al. 2021). As a result, occult metastasis remains common in clinically N0 early OSCC despite modern imaging (de la Fuente et al. 2023; Worthington et al. 2023; Adachi et al. 2026) and elective neck dissection has been shown to improve survival compared with observation and delayed treatment at relapse (D'Cruz et al. 2015), a finding since corroborated by meta-analytic and population-based data (He et al. 2025; Binda et al. 2026).

Beyond imaging accuracy and surgical strategy, accumulating evidence suggests that the time interval preceding treatment may itself influence disease progression and staging accuracy (Xiao et al. 2018; Singh et al. 2026). Consistent with this, prolonged time-to-surgery has repeatedly been linked to worse survival in head and neck cancer, including the oral cavity specifically (Rygalski et al. 2021).

Taken together, this literature establishes that delay across the broader diagnosis-to-treatment pathway can be associated with measurable disease progression and worse outcomes. However, this evidence largely concerns extended intervals (weeks to months) and overall clinical-to-pathologic stage migration, rather than the specific, comparatively short interval between cross-sectional imaging and surgery that is routine in most treatment pathways. Whether this narrower, clinically unavoidable interval is itself associated with the pathological nodal outcome, specifically in patients whose neck was initially staged as inconspicuous, has not been fully established (Koroglu et al. 2024). If occult nodal disease progresses measurably even within this short window, this would have direct implications for how a negative preoperative neck is interpreted and for the timing of surgical care. The present study therefore investigates whether the interval between computed tomography (CT) imaging and surgery (CT-to-surgery interval) is associated with the presence, extent, and extranodal extension of histopathologically confirmed lymph node metastases in a cohort of OSCC patients with clinical and radiologically unremarkable necks.

## Materials and methods

### Ethical considerations, study design and patient cohort

The study was conducted in accordance with the Declaration of Helsinki. Approval was obtained from the institutional ethics committee prior to data collection (approval number: 2022-234-S-KH). Due to the retrospective nature of the study and the use of anonymized data, the requirement for informed consent was waived.

**Fig. 1:**
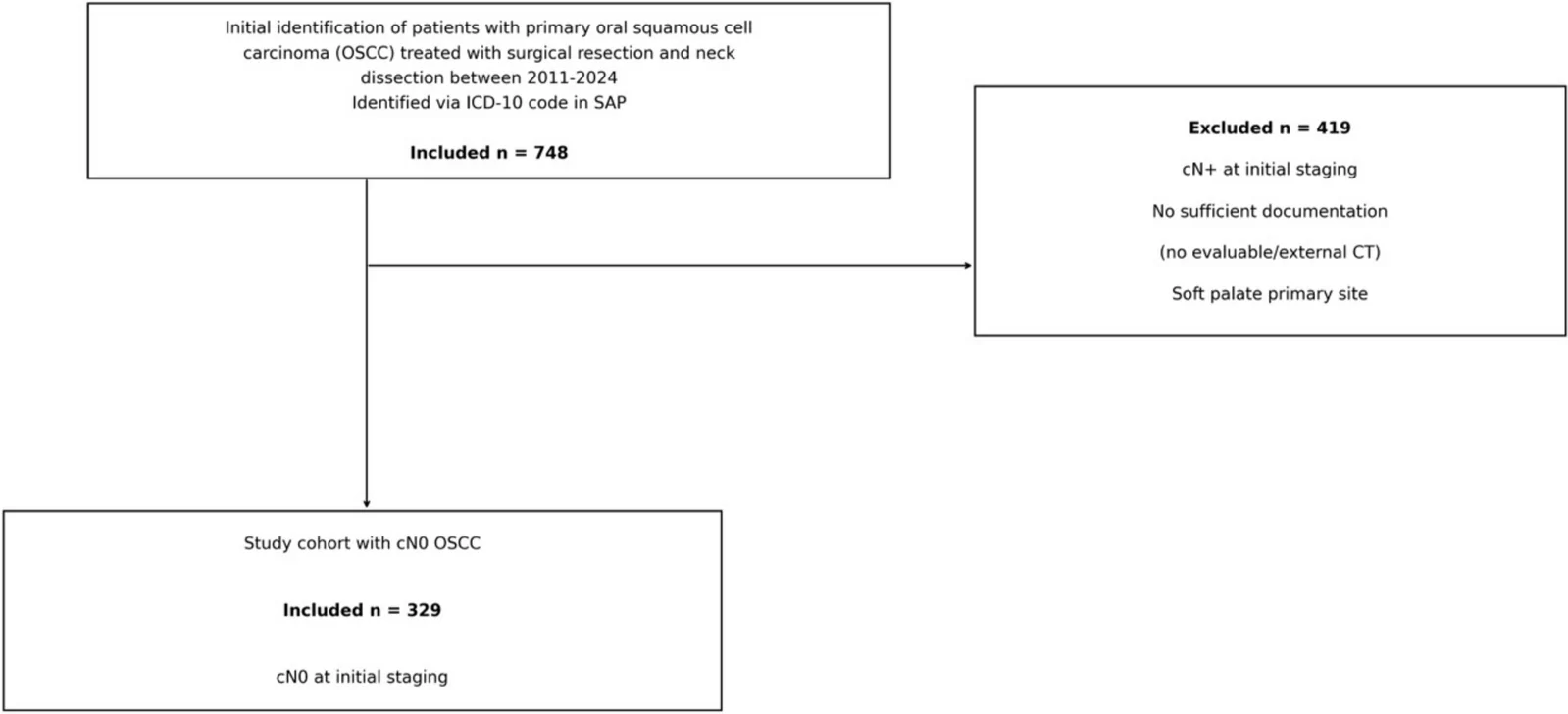
Patient selection flowchart. Of 748 patients with primary OSCC (2011–2024), 419 were excluded (recurrent/secondary tumor, non-evaluable or external CT, cN+ status, missing lymph node histology), yielding a final cN0 cohort of 329 patients

This retrospective single-center study included patients treated for primary OSCC between 2011 and 2024 in the Department of Oral and Maxillofacial Surgery, TUM University Hospital Klinikum Rechts der Isar, School of Medicine and Health, Technical University of Munich (Fig. 1). Inclusion criteria were availability of a preoperative, in-house performed contrast-enhanced CT scan of the head and neck, clinical nodal staging as cN0 based on the combined assessment of physical examination and CT imaging, definitive surgical resection of the primary tumor with neck dissection, and histopathological assessment of the resected lymph nodes. Patients with recurrent or secondary malignancies, clinically or radiologically suspicious lymph nodes (cN+) at initial staging, external CT imaging, missing histopathological nodal data were excluded.

### Radiological assessment

All preoperative CT examinations were reviewed based on the original radiology reports, extracted out of the hospital’s PACS (picture archiving and communication system), carried out by at least two board-certified radiologists with substantial experience in head and neck imaging. Radiological assessment of cervical lymph node status was categorized using an ordinal grading system:

- No suspicious lymph nodes
- Suspicious/metastatic lymph nodes

For the present analysis, only patients classified as radiologically inconspicuous (no suspicious findings) were included. The time interval between CT imaging and surgery was calculated in days for each patient.

### Histopathological assessment

Clinical and pathological data were extracted from the electronic patient records of the hospital information system. Histopathological evaluation of the surgical specimen and resected lymph nodes served as the reference standard. The following parameters were recorded: presence of histologically confirmed lymph node metastasis, number of positive lymph nodes, total number of lymph nodes removed, pathological N-stage, and presence of extranodal extension (ENE). Histopathological assessment was performed according to institutional standards by experienced head and neck pathologists.

### Tumor localization

Primary tumor localization was classified into the following anatomical subsites: floor of the mouth, tongue, mandibular alveolar ridge, maxillary alveolar ridge, buccal mucosa, retromolar region, and hard palate. Localization-specific analyses were performed to assess whether tumor site influenced the association between CT-to-surgery interval and nodal outcome.

### Statistical analysis

Descriptive statistics were used to summarize patient characteristics, tumor localization, and histopathological outcomes. Categorical variables are presented as frequencies and percentages, while continuous variables are reported as mean ± standard deviation or median with interquartile range, as appropriate.

The association between the CT-to-surgery interval and histopathologically confirmed lymph node metastasis and extranodal extension was assessed using the Mann–Whitney-U-test and, given violations of variance homogeneity, the Welch t-test. Logistic regression was used to evaluate the CT-to-surgery interval as a continuous predictor of lymph node positivity and extranodal extension, both unadjusted and adjusted for age and smoking status. Given overdispersion of the number of positive lymph nodes, negative binomial regression was used to assess the association between the CT-to-surgery interval and the number of positive lymph nodes, likewise unadjusted and adjusted for age and smoking status. Associations between tumor localization and both the CT-to-surgery interval and pathological outcomes were assessed using the chi-square test and the Kruskal–Wallis test, as appropriate, to evaluate localization as a potential confounder.

To assess the robustness of the primary findings, several sensitivity analyses were performed. The CT-to-surgery interval was categorized into quartiles and entered as a categorical predictor in logistic regression to evaluate the linearity of the dose–response relationship. The primary logistic regression was repeated after excluding patients with an interval exceeding 30 days, and the distribution of lymph node positivity within this excluded subgroup was examined using a cross-tabulation. To assess whether the observed associations were confounded by the extent of surgical lymph node yield, the logistic regression model for lymph node positivity was repeated with the number of lymph nodes removed as an additional covariate, and the negative binomial regression model for the number of positive lymph nodes was repeated using the number of lymph nodes removed as an offset. The association between the CT-to-surgery interval and the number of lymph nodes removed was assessed using Spearman’s rank correlation. Baseline characteristics (sex, age, ASA classification, smoking status, alcohol consumption, tumor localization, pathological T-stage, and bone infiltration, operative year) were additionally compared between patients with an interval exceeding 30 days and those with an interval of 30 days or less, using appropriate parametric or non-parametric tests, to assess for baseline confounding within this subgroup. In the multivariable negative binomial regression, inclusion of smoking status resulted in non-convergence due to quasi-separation; the adjusted model for the number of positive lymph nodes therefore includes age only.

A two-sided *p*-value <0.05 was considered statistically significant. All analyses were performed using IBM SPSS Statistics (version 29).

## Results

### Patient cohort and baseline characteristics

A total of 329 patients with primary OSCC and cN0 cervical lymph node status were included in the analysis (Fig. 1 and Table 1). The cohort comprised 204 men (62.0%) and 125 women (38.0%), with a median age of 63.1 years (IQR 55.0–72.4; mean 63.18 ± 12.58 years). Most patients were classified as ASA 2 (73.6%), followed by ASA 3 (18.8%) and ASA 1 (7.6%).

**Table 1:** Cohort characteristics

Parameter	n (%) / Median (IQR)
*Demographics*	
Sex	
Female	125 (38.0%)
Male	204 (62.0%)
Age [years]	63.1 (55.0–72.4)
ASA	
1	25 (7.6%)
2	242 (73.6%)
3	62 (18.8%)
*Risk factors*	
Smoking	
No	159 (48.3%)
Yes	170 (51.7%)
Alcohol	
No	240 (72.9%)
Yes	89 (27.1%)
*Tumor localization*	
Floor of mouth	130 (39.5%)
Tongue	101 (30.7%)
Alveolar ridge (mandible)	43 (13.1%)
Buccal mucosa	28 (8.5%)
Retromolar trigone	11 (3.3%)
Alveolar ridge (maxilla)	12 (3.6%)
*Hard palate*	4 (1.2%)
*Interval & pathological outcome*	
CT-to-surgery interval (days)	9.0 (5.0–15.0)
No. of LNs removed	26.0 (19.0–37.0)
Histologically positive LN	
No	276 (83.9%)
Yes	53 (16.1%)
No. of positive LNs	0.0 (0.0–0.0)
ENE	
No	316 (96.0%)
Yes	13 (4.0%)

Cohort characteristics of the study population (cN0 oral squamous cell carcinoma, n = 329). Values are presented as n (%) for categorical variables and median (interquartile range) for continuous variables

LN: lymph node, ENE: Extranodal extension, ASA: American society of anesthesiologists physical status classification

Regarding risk factors, 170 patients (51.7%) were smokers and 89 (27.1%) reported alcohol consumption. The most common OSCC site was the floor of the mouth (39.5%), followed by the tongue (30.7%), the mandibular alveolar ridge (13.1%), the buccal mucosa (8.5%), the maxillary alveolar ridge (3.6%), the retromolar trigone (3.3%), and the hard palate (1.2%).

The median CT-to-surgery interval was 9.0 days (IQR 5.0–15.0). A median of 26.0 lymph nodes (IQR 19.0–37.0) were removed per patient. Histopathologically confirmed lymph node metastasis was present in 53 of 329 patients (16.1%), with a median of 0 positive lymph nodes per patient across the whole cohort (IQR 0–0; mean 0.30 ± 0.95; range 0–9); among the 53 histologically node-positive patients, the median number of positive lymph nodes was 1 (IQR 1–2; mean 1.85 ± 1.65; range 1–9). Extranodal extension (ENE) was present in 13 patients (4.0%).

### Association between CT-to-surgery interval and lymph node positivity

The CT-to-surgery interval did not differ significantly between patients with and without histologically positive lymph nodes on Mann–Whitney-U-testing (*p* = 0.474) or on Welch t-test (mean 14.08 ± 13.80 vs. 11.04 ± 8.08 days, *p* = 0.127, Table 2**).**

**Table 2:** Association between the CT-to-surgery interval and pathological outcomes

Outcome	Test	Effect estimate (95% CI)	*p*-value
Histologically positive LN	Mann–Whitney-U (group comparison)		0.474
	Welch t-test	t(59.0) = −1.550	0.127
	Logistic regression, univariate	OR 1.031 (1.002–1.060)	0.035
	Logistic regression, multivariate	OR 1.027 (0.998–1.057)	0.065
No. of positive LNs	Spearman correlation	ρ = 0.059	0.287
	Negative binomial regression, univariate	IRR 1.026 (0.998–1.056)	0.068
	Negative binomial regression, adjusted (age only)	IRR 1.027 (0.998–1.056)	0.068
ENE	Mann–Whitney-U (group comparison)		0.732
	Welch t-test	t(12.8) = 0.133	0.896
	Logistic regression, univariate	OR 0.995 (0.935–1.059)	0.881
	Logistic regression, adjusted*	OR 0.989 (0.928–1.055)	0.740

Association between the CT-to-surgery interval and pathological outcomes in patients with cN0 oral squamous cell carcinoma (n = 329). Odds ratios (OR) and incidence rate ratios (IRR) refer to a one-day increase in the CT-to-surgery interval and were adjusted for age and smoking status, except for the negative binomial regression adjusted model, which was adjusted for age only because inclusion of smoking status resulted in non-convergence due to quasi-separation

CI: confidence interval, LN: lymph node, ENE: extranodal extension

**Fig. 2:**
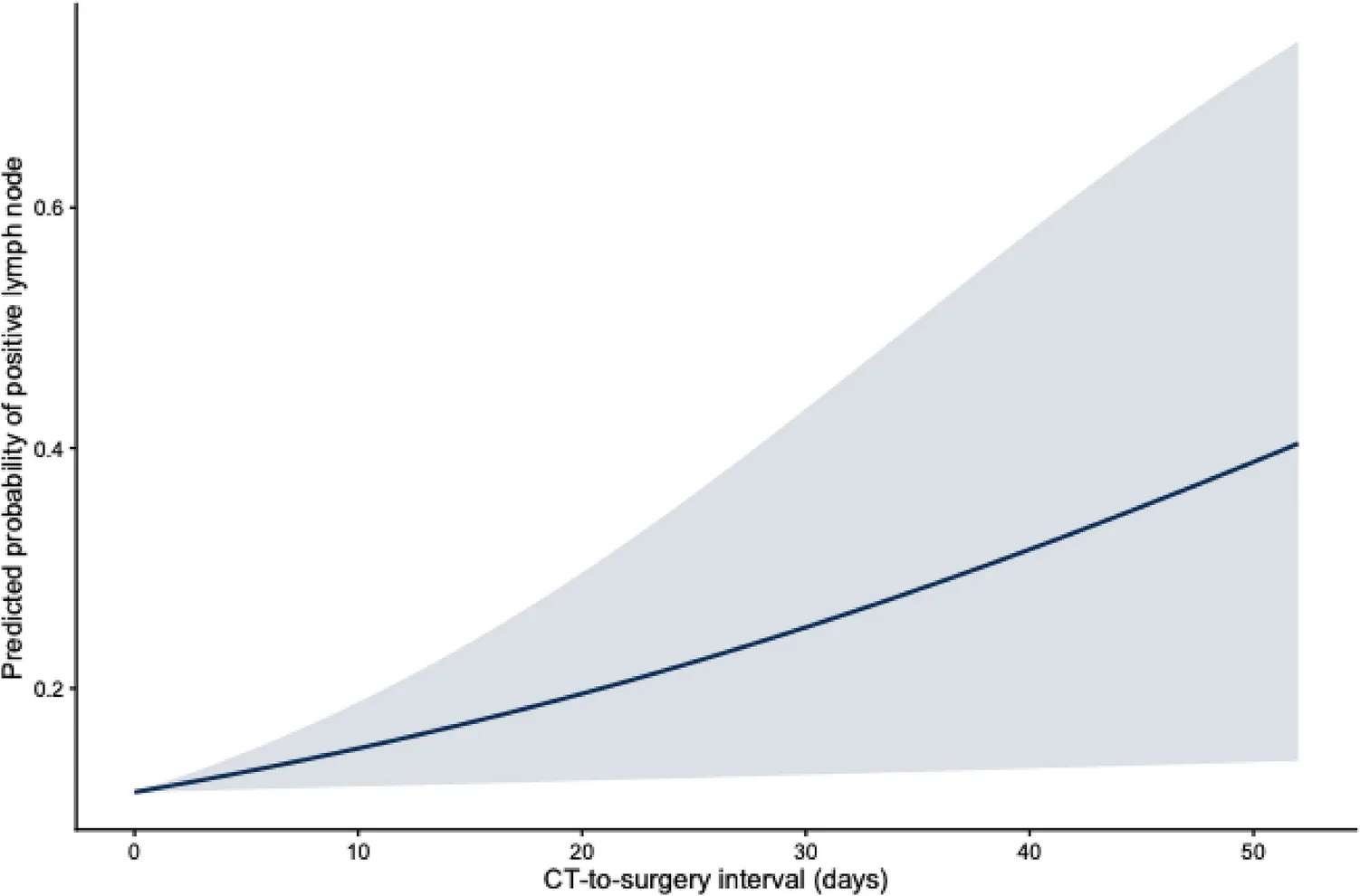
Predicted probability of histologically positive lymph nodes as a function of the CT-to-surgery interval, based on univariable logistic regression. The shaded area represents the 95% confidence interval

Univariable logistic regression treating the CT-to-surgery interval as a continuous predictor showed a significant association with lymph node positivity (OR 1.031, 95% CI 1.002–1.060, *p* = 0.035). In a multivariable model adjusted for age and smoking status, this association was attenuated and no longer reached statistical significance (OR 1.027, 95% CI 0.998–1.057, *p* = 0.065). The model-predicted probability of lymph node positivity across the observed interval range is shown in Fig. 2; however, as detailed below, this association was not robust across sensitivity analyses even before adjustment.

In sensitivity analyses, this association was not robust. When the CT-to-surgery interval was categorized into quartiles (Q1 ≤5 days, n = 87, 26.4%; Q2 6–9 days, n = 83, 25.2%; Q3 10–15 days, n = 79, 24.0%; Q4 >15 days, n = 80, 24.3%), no significant or monotonic trend was observed across categories relative to the shortest-interval quartile (omnibus *p* = 0.969; vs. Q1: Q2 OR 1.057, *p* = 0.896; Q3 OR 1.226, *p* = 0.628; Q4 OR 1.104, *p* = 0.816). After excluding the 13 patients (4.0%) with an interval exceeding 30 days, the association was fully attenuated (OR 0.995, 95% CI 0.952–1.040, *p* = 0.824). Within this subgroup of patients with an interval exceeding 30 days, lymph node positivity was notably more frequent than in the remainder of the cohort (53.8% vs. 14.6%). In a further exploratory sensitivity analysis additionally adjusting for the number of lymph nodes removed, the association between the CT-to-surgery interval and lymph node positivity was likewise attenuated toward the null (OR 1.015, *p* = 0.334), while the number of lymph nodes removed remained itself strongly associated with lymph node positivity. The CT-to-surgery interval showed a weak, non-significant positive correlation with the number of lymph nodes removed (Spearman’s ρ = 0.102, *p* = 0.063).

To further assess whether the markedly higher lymph node positivity rate observed among patients with an interval exceeding 30 days reflected baseline confounding rather than the interval itself, baseline characteristics were compared between this subgroup (n = 13) and the remaining patients with an interval of 30 days or less (n = 316; Table 3). Sex, ASA classification, smoking status, alcohol consumption, tumor localization, pathological T-stage, and bone infiltration did not differ significantly between the two groups, nor did age (median 60.0 vs. 63.1 years, *p* = 0.217). No temporal clustering by operative year was observed between the two groups. The number of lymph nodes removed was significantly higher among patients with an interval exceeding 30 days (median 37 vs. 25, *p* = 0.007). Pathological N-stage necessarily differed between the two groups, given its close correspondence with lymph node positivity itself, and was therefore not considered an informative baseline comparison. Depth of invasion was not compared between groups because of near-complete missingness in the >30-day subgroup (recorded in only 2 of 13 patients).

**Table 3:** Baseline characteristics: ≤30-day versus >30-day CT-to-surgery interval

Variable	≤30 days (n = 316)	>30 days (n = 13)	*p*-value
Sex (% male)	61.1%	84.6%	0.087
ASA (1/2/3, %)	7.6 / 73.4 / 19.0	7.7 / 76.9 / 15.4	0.948
Smoking (% yes)	50.6%	76.9%	0.063
Alcohol (% yes)	n.s	n.s	0.345
Tumor site, floor of mouth (%)	38.3%	69.2%	0.075
Pathological T-stage	predominantly T1–T2	predominantly T1–T2	0.402
Bone infiltration (% yes)	14.4%	23.1%	0.386
Age [years], median	63.1	60.0	0.217
No. of LNs removed (yield), median	25	37	0.007
Operative year, median	2015	2014	0.177

Comparison of baseline characteristics between patients with a CT-to-surgery interval exceeding 30 days (n = 13) and patients with an interval of 30 days or less (n = 316). N-stage was not compared because it corresponds closely to lymph node positivity itself; depth of invasion was not compared because of near-complete missingness in the >30-day subgroup

ASA: American society of anesthesiologists physical status classification, LN: lymph node

### Association between CT-to-surgery interval and the number of positive lymph nodes

The number of histologically positive lymph nodes per patient was median 0 (IQR 0–0; mean 0.30 ± 0.95; range 0–9) across the whole cohort; among the 53 histologically node-positive patients, the median number of positive lymph nodes was 1 (IQR 1–2; mean 1.85 ± 1.65; range 1–9). The Spearman correlation between the CT-to-surgery interval and the number of positive lymph nodes was weak and not statistically significant (ρ = 0.059, *p* = 0.287).

Negative binomial regression showed a positive but non-significant association between the CT-to-surgery interval and the number of positive lymph nodes in the univariable model (IRR 1.026, 95% CI 0.998–1.056, *p* = 0.068). Because inclusion of smoking status resulted in non-convergence due to quasi-separation, the multivariable model was adjusted for age only, and likewise did not reach statistical significance (IRR 1.027, 95% CI 0.998–1.056, *p* = 0.068). In a sensitivity analysis using the number of lymph nodes removed as an offset, this association remained weak and non-significant (IRR 1.019, *p* = 0.135).

### Extranodal extension

Extranodal extension was present in 13 of 329 patients (4.0%). The CT-to-surgery interval showed no significant association with ENE on Mann–Whitney-U-testing (*p* = 0.732), Welch t-test (mean 11.15 ± 10.48 vs. 11.55 ± 9.25 days, *p* = 0.896), univariable logistic regression (OR 0.995, 95% CI 0.935–1.059, *p* = 0.881), or the multivariable model adjusted for age and smoking status (OR 0.989, 95% CI 0.928–1.055, *p* = 0.740).

### Role of tumor localization

Tumor localization was not significantly associated with histopathological lymph node positivity (χ^2^ = 8.971, df = 6, *p* = 0.175). In contrast, tumor localization was significantly associated with ENE (χ^2^ = 23.972, df = 6, *p* < 0.001); however the elevated ENE rates were concentrated in small subgroups (retromolar trigone 27.3%, 3/11; hard palate 25.0%, 1/4; buccal mucosa 7.1%, 2/28; tongue 4.0%, 4/101; alveolar ridge (mandible) 2.3%, 1/43; floor of mouth 1.5%, 2/130; alveolar ridge (maxilla) 0%, 0/12); this finding should therefore be interpreted as hypothesis-generating rather than as evidence of a robust localization effect. The CT-to-surgery interval did not differ significantly across tumor subsites, although the result approached the conventional significance threshold (Kruskal–Wallis H = 11.942, df = 6, *p* = 0.063). In a multivariable logistic regression model including both the CT-to-surgery interval and tumor localization, the interval remained associated with lymph node positivity (OR 1.032, *p* = 0.031); localization was not significant (*p* = 0.105).

## Discussion

In this cohort of 329 patients with cN0 oral cavity OSCC, the CT-to-surgery interval was associated with histopathologically confirmed lymph node positivity in univariable analysis, but this association was attenuated after adjustment for age and smoking status and was not robust across sensitivity analyses. Importantly, the data did not indicate a continuous increase in occult nodal positivity across the range of CT-to-surgery intervals typically encountered in clinical practice. Instead, the observed association appeared to be driven primarily by a small subgroup of patients with intervals exceeding 30 days.

Patients with intervals exceeding 30 days had a markedly higher rate of occult nodal metastasis than those undergoing surgery within 30 days (53.8% vs. 14.6%). However, only 13 patients belonged to this subgroup, limiting the reliability of this observation. The measured patient, tumor, and study-period characteristics did not differ significantly between patients with intervals >30 and ≤30 days, including age, sex, ASA classification, smoking and alcohol consumption, tumor localization, pathological T-stage, bone infiltration, and year of surgery. Nevertheless, the small subgroup size does not exclude residual confounding. Moreover, the specific reasons for prolonged surgical delay were not systematically documented. The high rate of nodal positivity among patients with intervals exceeding 30 days should therefore be regarded as hypothesis-generating rather than evidence of a biological threshold effect.

Lymph node yield deserves separate consideration. Patients with intervals exceeding 30 days had a higher median number of removed lymph nodes than those with shorter intervals (37 vs. 25, *p* = 0.007). However, the overall correlation between CT-to-surgery interval and lymph node yield was weak and did not reach statistical significance (Spearman’s ρ = 0.102, *p* = 0.063). Because lymph node yield is determined during surgery and may itself influence the probability of detecting occult metastases, its role as a confounder is uncertain. Accordingly, attenuation of the interval–outcome association after adjustment for lymph node yield should be interpreted cautiously and does not establish a mediating effect.

The occult metastasis rate of 16.1% observed in the present cohort lies at the lower end of previously reported ranges, which commonly approximate 20–30% and reach up to 43% in selected series (de la Fuente et al. 2023; Worthington et al. 2023; Adachi et al. 2026) while more recent surgical cohorts have reported rates of 16.9–25.2% (Duvernay et al. 2025; Neuhaus et al. 2026). Such occult disease is consistent with the known limitations of radiological staging of the clinically negative neck, particularly for small metastatic deposits (Liao et al. 2012). The present study therefore addresses a clinically relevant question: whether the time elapsed after a negative staging CT contributes measurably to the probability of identifying nodal disease at surgery.

Previous studies support the biological plausibility of tumor progression during treatment delays. Xiao et al. (2018) demonstrated an association between increasing time to treatment initiation and clinical-to-pathological upstaging in a large head and neck cancer cohort. More recently, Singh et al. (2026) prospectively demonstrated measurable OSCC progression during the pretreatment period, with a median tumor volume doubling time of 7.9 weeks and radiographic nodal progression in 23% of patients. However, these studies evaluated substantially broader treatment intervals, generally spanning several weeks, and addressed overall tumor or stage progression rather than the specific interval between staging CT and surgery in patients with a radiologically negative neck. The present cohort therefore examines a considerably narrower time window, with a median CT-to-surgery interval of only 9 days.

No association was observed between the CT-to-surgery interval and extranodal extension. Given the low prevalence of ENE in this cN0 cohort (4.0%), however, this analysis was likely underpowered. Tumor localization was likewise not associated with lymph node positivity or the CT-to-surgery interval and did not materially alter the interval–outcome association. Although localization was significantly associated with ENE, this finding was based on few events distributed across several small subgroups and should therefore be interpreted cautiously. Floor of mouth (39.5%) and tongue (30.7%) were the predominant primary sites in the cohort. No subsite-specific selection criteria were applied apart from the predefined exclusion of soft-palate tumors, and the observed distribution therefore reflects the eligible institutional cohort rather than deliberate case selection. Although previous studies have reported differences in occult metastatic risk according to primary tumor site (Eskander et al. 2024; Neuhaus et al. 2026), localization was not identified as a relevant confounder of the interval–outcome association in the present study.

Several limitations should be considered. The retrospective, single-center design precluded assessment of between-institution differences and limits causal inference and generalizability, and the reasons for prolonged surgical delay were not systematically documented. Clinical T-stage was not consistently available, and residual confounding by tumor biology or surgical complexity cannot be excluded. Depth of invasion and pathological T-stage, although established predictors of occult metastasis (Alqutub et al. 2024), were not included as primary preoperative covariates because they were determined from the surgical specimen. No patient received neoadjuvant therapy before surgery. Importantly, the size and histological classification of occult nodal deposits as isolated tumor cells, micrometastases, or macrometastases were not recorded. It therefore remains impossible to determine whether metastases detected at surgery developed during the CT-to-surgery interval or were already present below the detection threshold of CT at initial staging. The small number of patients with intervals exceeding 30 days and the low number of ENE events further limit subgroup analyses. Finally, the findings relate specifically to CT-based staging and may not directly translate to MRI- or PET/CT-based staging pathways with different diagnostic performance (Guedj et al. 2024).

## Conclusion

In patients with cN0 OSCC, the CT-to-surgery interval was not robustly associated with occult nodal metastasis across the range typically encountered in routine clinical practice. These findings provide some reassurance regarding modest, unavoidable scheduling-related intervals between staging and surgery and may help inform counseling of patients concerned about short treatment delays. However, the implications of substantially prolonged intervals remain uncertain and should be investigated in larger cohorts with detailed information on the reasons for treatment delay.

## Data Availability

The datasets generated during and analysed during the current study are available from the corresponding author on reasonable request.

## References

[CR1] Adachi M, Ohta S, Ishii A, Motohashi M (2026) Contemporary management of the clinically N0 neck in oral squamous cell carcinoma. Oral Oncol 174:107839 10.1016/j.oraloncology.2025.107839 41554216

[CR2] Alqutub S, Alqutub A, Bakhshwin A, Mofti Z, Alqutub S, Alkhamesi AA, Nujoom MA, Rammal A, Merdad M, Marzouki HZ (2024) Histopathological predictors of lymph node metastasis in oral cavity squamous cell carcinoma: a systematic review and meta-analysis. Front Oncol 14:1401211 10.3389/fonc.2024.1401211 PMC11148647 38835393

[CR3] Binda NC, Lavareze L, Vieira GS, Tincani PDC, Tincani AJ, Chone CT, Altemani A, Mariano FV (2026) Impact of elective cervical dissection on the prognosis of patients with oral squamous cell carcinoma cT1/T2N0: a systematic review and meta-analysis. Crit Rev Oncol Hematol 217:104982 10.1016/j.critrevonc.2025.104982 41115617

[CR4] D’Cruz AK, Vaish R, Kapre N, Dandekar M, Gupta S, Hawaldar R, Agarwal JP, Pantvaidya G, Chaukar D, Deshmukh A, Kane S, Arya S, Ghosh-Laskar S, Chaturvedi P, Pai P, Nair S, Nair D, Badwe R (2015) Head, neck disease management g: elective versus therapeutic neck dissection in node-negative oral cancer. N Engl J Med 373:521–529 10.1056/NEJMoa1506007 26027881

[CR5] de la Fuente C, Prat-Valero N, Alberola-Ferranti M, Mis-Castell D, Saez-Barba M, Pujol-Pina R, Pamias-Romero J, Bescos-Atin C (2023) Occult metastases of oral maxillary squamous cell carcinoma: systematic review and meta-analysis. Head Neck 45:733–744 10.1002/hed.27276 36515647

[CR6] Duvernay J, Garreau B, Dubreuil PA, Bondaz M, Majoufre C, Schlund M (2025) Lymph node metastasis in level IIb neck dissection for clinically node-negative oral squamous cell carcinoma patients: an 11-year retrospective study. Int J Oral Maxillofac Surg 54:577–580 10.1016/j.ijom.2024.12.004 39753461

[CR7] Ekanayaka RP, Tilakaratne WM (2025) Impact of histopathological parameters in prognosis of oral squamous cell carcinoma. Oral Dis 31:1420–1438 10.1111/odi.15035 38938003

[CR8] Eskander A, Dziegielewski PT, Patel MR, Jethwa AR, Pai PS, Silver NL, Sajisevi M, Sanabria A, Doweck I, Khariwala SS, St John M (2024) Oral cavity cancer surgical and nodal management: a review from the American Head and Neck Society. JAMA Otolaryngol Head Neck Surg 150:172–178 10.1001/jamaoto.2023.4049 38153725

[CR9] Guedj D, Neveu S, Becker M, Mermod M (2024) FDG PET-CT for the detection of occult nodal metastases in head and neck cancer: a systematic review and meta-analysis. Cancers (Basel). 10.3390/cancers16172954 PMC11394031 39272812

[CR10] He Y, Shui H, Liu J, He Y, Wu J, Chen Q (2025) Neck dissection improves the prognosis of patients with early-stage oral squamous cell carcinoma. Sci Rep 15:11640 10.1038/s41598-025-96018-2 PMC11971466 40185843

[CR11] Horvath A, Prekopp P, Polony G, Szekely E, Tamas L, Danos K (2021) Accuracy of the preoperative diagnostic workup in patients with head and neck cancers undergoing neck dissection in terms of nodal metastases. Eur Arch Otorhinolaryngol 278:2041–2046 10.1007/s00405-020-06324-w PMC8131330 32860560

[CR12] Johansson G, Hysek MH, Marklund L, Bark R, Piersiala K (2026) Prognostic impact of micrometastases and nodal status in early and advanced oral squamous cell carcinoma. Eur Arch Otorhinolaryngol 283:3305–3316 10.1007/s00405-026-10070-w PMC13152956 41786952

[CR13] Ketschau J, Mohr J, Grabenhorst A, Singer H, Kram H, Leopold C, Krautkremer N, Pippich K, Wolff KD, Ritschl LM (2026) From nodal metastasis to oncologic recurrence in tongue and oral floor squamous cell carcinoma: distinct failure patterns and clinicopathological risk profiles. Head Neck. 10.1002/hed.7034942266014

[CR14] Koroglu E, Sirin S, Isgoren S (2024) The importance of the time interval between preoperative 18F-FDG PET/CT imaging and neck dissection for the detection of nodal metastases in patients with head and neck squamous cell carcinoma. Niger J Clin Pract 27:859–864 10.4103/njcp.njcp_38_24 39082911

[CR15] Liao LJ, Lo WC, Hsu WL, Wang CT, Lai MS (2012) Detection of cervical lymph node metastasis in head and neck cancer patients with clinically N0 neck-a meta-analysis comparing different imaging modalities. BMC Cancer 12:236 10.1186/1471-2407-12-236 PMC3476985 22691269

[CR16] Neuhaus MT, Weniger G, Papazacharias E, Fenske F, Jehn P, Lentge F, Korn P, Gellrich NC, Zimmerer R (2026) Cervical lymph node metastasis patterns and diagnostic accuracy of preoperative staging in oral squamous cell carcinoma. Cancers (Basel). 10.3390/cancers18111851 PMC13257117 42279433

[CR18] Rygalski CJ, Zhao S, Eskander A, Zhan KY, Mroz EA, Brock G, Silverman DA, Blakaj D, Bonomi MR, Carrau RL, Old MO, Rocco JW, Seim NB, Puram SV, Kang SY (2021) Time to surgery and survival in head and neck cancer. Ann Surg Oncol 28:877–885 10.1245/s10434-020-09326-4 PMC7664584 33188461

[CR19] Sandulache VC, Michikawa C, Kataria P, Gleber-Netto FO, Bell D, Trivedi S, Rao X, Wang J, Zhao M, Jasser S, Myers JN, Pickering CR (2018) High-risk TP53 mutations are associated with extranodal extension in oral cavity squamous cell carcinoma. Clin Cancer Res 24:1727–1733 10.1158/1078-0432.CCR-17-0721 PMC5884733 29330202

[CR20] Singh AG, Mahajan A, Sinha S, Shetty R, Mohanty S, Kumar A, Chakrabarty N, Joshi P, Nair S, Laskar SG, Prabhash K, Chaturvedi P (2026) Tumour growth kinetics and stage progression during treatment delay in oral squamous cell carcinoma. Oral Oncol 179:108033 10.1016/j.oraloncology.2026.108033 42190340

[CR21] Struckmeier AK, Buchbender M, Lutz R, Agaimy A, Kesting M (2024) Comparison of the prognostic value of lymph node yield, lymph node ratio, and number of lymph node metastases in patients with oral squamous cell carcinoma. Head Neck 46:1083–1093 10.1002/hed.27748 38501325

[CR22] Worthington HV, Bulsara VM, Glenny AM, Clarkson JE, Conway DI, Macluskey M (2023) Interventions for the treatment of oral cavity and oropharyngeal cancers: surgical treatment. Cochrane Database Syst Rev 8:CD006205 10.1002/14651858.CD006205.pub5 PMC10476948 37650478

[CR23] Xiao R, Ward MC, Yang K, Adelstein DJ, Koyfman SA, Prendes BL, Burkey BB (2018) Increased pathologic upstaging with rising time to treatment initiation for head and neck cancer: a mechanism for increased mortality. Cancer 124:1400–1414 10.1002/cncr.31213 29315499

